# Beyond Trisomy 21: Additional Chromosomal Anomalies Detected through Routine Aneuploidy Screening

**DOI:** 10.3390/jcm3020388

**Published:** 2014-04-08

**Authors:** Amy Metcalfe, Catriona Hippman, Melanie Pastuck, Jo-Ann Johnson

**Affiliations:** 1 Department of Obstetrics and Gynaecology, University of British Columbia, BC Women’s Hospital 4500 Oak St., Vancouver, BC V6H 3N1, Canada; 2 Department of Psychiatry, University of British Columbia, Vancouver, BC, Canada; E-Mail: catriona.hippman@ubc.ca; 3 Women’s Health Research Institute, BC Women’s Hospital, 4500 Oak St., Vancouver, BC V6H 3N1, Canada; 4 Early Risk Assessment Program, Suite 100 3820 Hospital Dr NW, Calgary, AB T2N 4N1, Canada; E-Mail: melanie.pastuck@albertahealthservices.ca; 5 Department of Obstetrics and Gynaecology, Foothills Medical Centre—North Tower, University of Calgary, 1403 29th St. NW, Calgary, AB T2N 2T9, Canada; E-Mail: joann.johnson@albertahealthservices.ca

**Keywords:** aneuploidy, prenatal, maternal serum, ultrasound, non-invasive testing, sensitivity, specificity

## Abstract

Prenatal screening is often misconstrued by patients as screening for trisomy 21 alone; however, other chromosomal anomalies are often detected. This study aimed to systematically review the literature and use diagnostic meta-analysis to derive pooled detection and false positive rates for aneuploidies other than trisomy 21 with different prenatal screening tests. Non-invasive prenatal testing had the highest detection (DR) and lowest false positive (FPR) rates for trisomy 13 (DR: 90.3%; FPR: 0.2%), trisomy 18 (DR: 98.1%; FPR: 0.2%), and 45,X (DR: 92.2%; FPR: 0.1%); however, most estimates came from high-risk samples. The first trimester combined test also had high DRs for all conditions studied (trisomy 13 DR: 83.1%; FPR: 4.4%; trisomy 18 DR: 91.9%; FPR: 3.5%; 45,X DR: 70.1%; FPR: 5.4%; triploidy DR: 100%; FPR: 6.3%). Second trimester triple screening had the lowest DRs and highest FPRs for all conditions (trisomy 13 DR: 43.9%; FPR: 8.1%; trisomy 18 DR: 70.5%; FPR: 3.3%; 45,X DR: 77.2%; FPR: 9.3%). Prenatal screening tests differ in their ability to accurately detect chromosomal anomalies. Patients should be counseled about the ability of prenatal screening to detect anomalies other than trisomy 21 prior to undergoing screening.

## 1. Introduction

Prenatal screening for fetal aneuploidy aims to identify women at increased risk of carrying a fetus with a chromosomal anomaly and limit the offer of invasive diagnostic tests (with their associated risk of miscarriage) to those women at high-risk. National practice guidelines currently recommend that all pregnant women be offered prenatal screening for aneuploidy [1,2]. Methods for prenatal screening have evolved rapidly in recent decades from screening based on maternal age alone, to serum screening, to a combination of maternal serum and ultrasound based measures, and most recently to isolated cell free fetal DNA (cffDNA) from maternal plasma [3,4,5].

Prenatal screening for fetal aneuploidy is most frequently discussed in terms of prenatal screening for trisomy 21 (Down syndrome) as this is the most common form of fetal aneuploidy and this condition has clinical implications for the health of the fetus [3,4]. However, counseling issues arise when anomalies are detected that were not discussed during the informed consent process, given that parents would be unprepared for such a result. Parents consistently report that they do not have enough information about prenatal screening, and may be surprised when they undergo a screening test for trisomy 21 only to find out that their fetus has another (and potentially more severe) anomaly [6,7,8,9]. A good understanding of prenatal screening, prior to accessing this test, is important, because if the results are positive, parents must make important and complex decisions about invasive testing which carries a small, but substantial risk of pregnancy loss, and potentially pregnancy termination [9,10]. Counseling about conditions other than trisomy 21 that may be detected through prenatal screening is complicated for a variety of reasons; the clinical implications of balanced chromosome rearrangements are not always known, the risk of specific anomalies is extremely low, and the reported detection rates and false positive rates for different anomalies vary widely in the literature [10]. This study aimed to systematically review the literature and use diagnostic meta-analysis to derive pooled detection and false positive rates for fetal aneuploidies other than trisomy 21 using standard prenatal screening tests. This information may be helpful to care providers as they discuss the relative strengths and limitations of different forms of prenatal screening with their patients.

## 2. Methods

### 2.1. Systematic Review

Relevant English-language literature was identified through a systemic search of Medline (1946–2013) and Embase (1974–2013) in November 2013. Reference lists of included articles were examined to identify additional relevant articles that may have been missed in the electronic search. The search terms included generic terms (prenatal diagnosis, antenatal diagnosis, prenatal screening, antenatal screening) as well as specific terms related to the screening test used (first trimester screening, aneuploidy screening, integrated screening, sequential screening, non-invasive prenatal testing, serum screening, combined screening, genetic screening, quad screen) or the chromosomal anomaly (Klinefelter syndrome, tetraploidy, translocation, triploidy, trisomy 13, trisomy 18, Turner syndrome). Truncation symbols were used to include all possible variations of the search term (*i.e.*, screen, screening, screened).

Studies were eligible for inclusion if they provided the data necessary to calculate the number of true positives, false positives, false negatives and true negatives for a chromosomal anomaly other than trisomy 21 using a currently available prenatal screening test (see Table 1). Studies that exclusively used maternal serum alpha fetoprotein (MS-AFP) or a combination of MS-AFP and human chorionic gonadotrophin (hCG) (double test) were deemed outdated and were not included in the review. Two reviewers (Amy Metcalfe, Catriona Hippman) independently reviewed all titles and abstracts for potential inclusion. Full-text review was undertaken for any article deemed potentially eligible by either reviewer. Data extraction and verification was performed by the same reviewers. Specific study elements extracted included: screening test used, detection rate, number of women screened, number of screen positive women, number of aneuploid fetuses, study location, and time period of data collection.

**Table 1:** Prenatal screening tests eligible for inclusion in systematic review.

Prenatal Screening Test	Biochemical and Ultrasound Components
First Trimester Combined Test	NT, PAPP-A, free β hCG
Second Trimester Triple Screen	AFP, hCG, uE3
Second Trimester Quadruple Screen	AFP, hCG, uE3, Inhibin A
Integrated/Sequential/Contingent Screen	(NT, PAPP-A, free β hCG) + (AFP, hCG, uE3, Inhibin A)
Non-Invasive Prenatal Testing (NIPT)	cffDNA

AFP = alpha-fetoprotein; cffDNA = cell free fetal DNA; hCG = human chorionic gonadatrophin; NT = nuchal translucency; PAPP-A = pregnancy associated plasma protein A; uE3 = unconjugated estriol.

Guidelines for the Meta-analysis of Observational Studies in Epidemiology (MOOSE) [11] and Studies of Diagnostic Accuracy (STARD) [12] were followed.

### 2.2. Diagnostic Meta-Analysis

The accuracy of prenatal screening tests is typically evaluated using both the detection rate (sensitivity) and the false positive rate (1-specificity). As these measures are correlated, a bivariate random effects model was used to derive pooled estimates and generate summary receiver operating characteristic (SROC) curves [13]. Studies were weighted based on their total sample size, as large sample sizes allow for more precise estimates of sensitivity and specificity [14]. For a detailed description of the technical specifications of the model, readers are referred to [14]. A minimum of four studies were required to derive pooled detection and false positive rates. All analyses were conducting using Stata 12 SE (StataCorp LP, College Station, TX, USA).

## 3. Results

**Figure 1:**
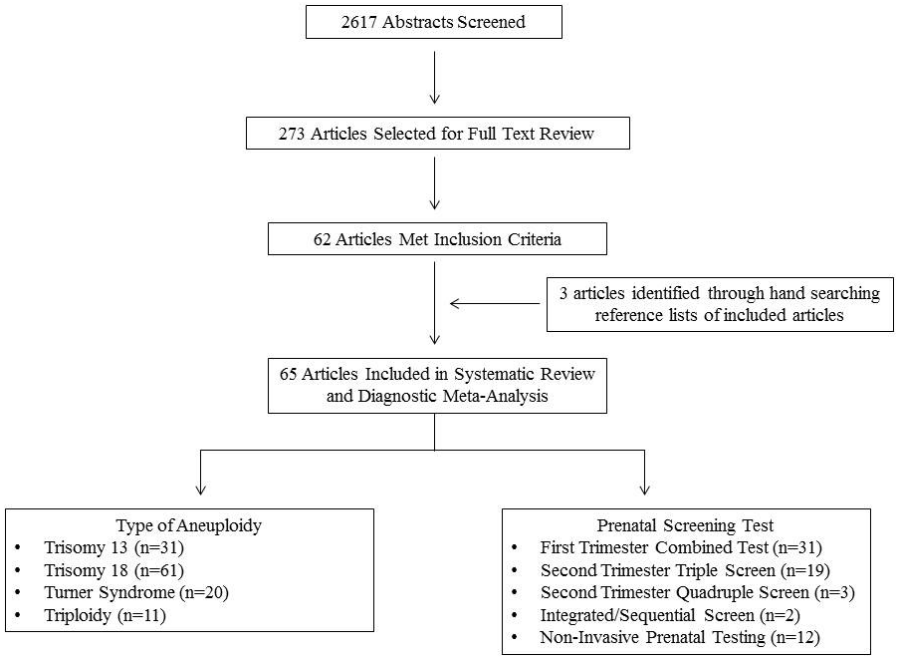
Flow diagram of study selection.

Sixty-five articles met all inclusion criteria and were included in the systematic review (Figure 1). Trisomy 18 was the most common aneuploidy eligible for inclusion in the meta-analysis for which literature was available, while the first trimester combined test was the most common prenatal screening test eligible for inclusion in the meta-analysis for which literature was available (Figure 1). Detailed information on all included studies can be found in the Appendix A.

### 3.1. Trisomy 13 (Patau Syndrome)

Overall, 31 studies reported data on trisomy 13, including 16 studies that utilized the first trimester combined test [15,16,17,18,19,20,21,22,23,24,25,26,27,28,29,30], 8 utilizing the second trimester triple screen [31,32,33,34,35,36,37,38], and 9 using non-invasive prenatal testing (NIPT) [39,40,41,42,43,44,45] (Table 2 and Appendix B). Two studies utilizing NIPT [39,45] included data on two patient groups (a training set and a validation set); hence were included as two distinct studies in the diagnostic meta-analysis.

Substantial variation was noted in the definition of a “screen positive” test for trisomy 13 using the first trimester combined test—definitions for specific risk levels for trisomy 13 included risks ≥1/200 [19], risks of trisomy 13 or 18 ≥1/150 [15,21] or 1/200 [25], or risks of trisomy 13, 18 or 21 ≥1/250 [24] or 1/300 [23,27]. No studies using the second trimester triple screen included a specific risk algorithm for trisomy 13; however, variation in the definition of “screen positive” for trisomy 21 or trisomy 21/trisomy 18 was observed. Studies differed in that some reported cut-off values based on the risk of trisomy 13 at the time of the test (either the first trimester or the second trimester) or at term; however, this only partially explains the differences observed in cut-off values.

NIPT exhibited the strongest overall test performance with a pooled detection rate of 90.3% and a false positive rate <1% (Table 2). The first trimester combined test had a significantly higher detection rate (83.1% *vs.* 43.9%) and a lower false positive rate (4.4% *vs.* 8.1%) than the second trimester triple screen, although statistical significance was not achieved for the false positive rate (*p* > 0.05) (Table 2).

**Table 2:** Pooled results for trisomy 13.

Study Characteristics	Prenatal Screening Test		
	1st Trimester Combined Test	2nd Trimester Triple Screen	Non-Invasive Prenatal Testing
Number of Studies	16	8	9
Number of Patients	245,502	1,276,894	5840
Number of Cases	185	156	86
Median prevalence per 10,000 pregnancies (25th–75th percentile range)	5.1 (3.8–16.0)	2.2 (1.2–9.0)	263.2 (71.1–637.8)
Observed Detection Rates from Included Studies (Minimum-Maximum Range)	0%–100%	0%–100%	63.6%–100%
Observed False Positive Rates from Included Studies (Minimum-Maximum Range)	0.1%–12.5%	0.2%–26.1%	0%–1.6%
Pooled Detection Rate	83.1% (72.6–90.2)	43.9% (23.0–67.2)	90.3% (75.7–96.6)
Pooled False Positive Rate	4.4% (3.0–6.4)	8.1% (3.1–19.7)	0.2% (0.05–0.8)

### 3.2. Trisomy 18 (Edward Syndrome)

Trisomy 18 was the most frequently reported condition in eligible studies. Data was obtained from 61 studies, including 30 that utilized the first trimester combined test [15,16,17,18,19,20,21,23,24,25,26,27,28,29,30,46,47,48,49,50,51,52,53,54,55,56,57,58,59,60], 17 that used the second trimester triple screen [31,32,33,34,35,36,37,38,61,62,63,64,65,66,67,68,69], 12 that contained data on NIPT [40,41,42,43,44,45,70,71,72,73,74], 3 studies that used the second trimester quadruple screen [47,75,76], and 2 studies that reported data on patients undergoing integrated or sequential screening [52,77] (Table 3 and Appendix B). One of the studies reporting on NIPT included data from two separate patient groups [72], and is included as two separate studies in the diagnostic meta-analysis.

Similar to screening for trisomy 13, substantial heterogeneity was observed in the definition for a “screen positive” test for trisomy 18. Positive risk scores for the first trimester combined screen ranged from risks ≥1/100 to ≥1/300. Approximately half of the studies using the second trimester triple screen that reported their definition for “screen positive” results used a patient-specific risk (risks ≥1/100 or ≥1/200), while the remaining studies used a fixed cut-off level based on levels of serum analytes (typically AFP ≤ 0.75 MoM, hCG ≤ 0.55 MoM, and uE3 ≤ 0.60 MoM). Again, studies differed in that some reported cut-off values based on the risk of trisomy 18 at the time of the test (either the first trimester or the second trimester) or at term.

**Table 3:** Pooled results for trisomy 18.

Study Characteristics	Prenatal Screening Test		
	1st Trimester Combined Test	2nd Trimester Triple Screen	Non-Invasive Prenatal Testing
Number of Studies	30	17	12
Number of Patients	325,808	1,752,184	10,778
Number of Cases	581	693	302
Median prevalence per 10,000 pregnancies (25th–75th percentile range)	13.9 (8.3–21.3)	4.8 (3.0–22.5)	454.2 (134.8–801.3)
Observed Detection Rates from Included Studies (Minimum-Maximum Range)	50%–100%	0%–100%	90%–100%
Observed False Positive Rates from Included Studies (Minimum-Maximum Range)	0.4%–15.8%	0.2%–35.7%	0%–2.0%
Pooled Detection Rate	91.9% (85.8–95.6)	70.5% (60.9–78.6)	98.1% (95.1–99.2)
Pooled False Positive Rate	3.5% (2.5–4.9)	3.3% (3.1–3.6)	0.2% (0.1–0.4)

NIPT exhibited the best overall test performance with a pooled detection rate of 98.1% and false positive rate of <1% (Table 3). The first trimester combined test outperformed the second trimester triple screen, as it had a significantly higher detection rate (91.9% *vs.* 70.5%), with no difference in the false positive rate (*p* > 0.05) (Table 3). There were an insufficient number of studies to derive pooled estimates on the detection rate and false positive rate for trisomy 18 using the second trimester quadruple test, but the observed detection rate ranged from 44.4% to 100% with false positive rates ranging from 0.5% to 9.6% [47,75,76]. Pooled results from the triple screen population may be generalizable to women screened with the quadruple test, as Inhibin A is not part of the trisomy 18 screening protocol.

There were an insufficient number of studies using integrated/sequential screening to determine pooled detection and false positive rates; however, the observed detection rate was 100% in both studies using integrated/sequential screening, while the observed false positive rates ranged from 3.7% to 7.3% [52,77].

### 3.3. 45,X (Turner Syndrome)

Twenty studies included data on Turner syndrome, 6 that utilized the first trimester combined screen [19,23,26,30,49,51], 9 that used the second trimester triple screen [31,32,33,34,35,36,66,78,79], 4 that involved NIPT [40,42,43,45], and a single study that used the second trimester quadruple screen [76] (Table 4 and Appendix B).

**Table 4:** Pooled results for 45,X.

Study Characteristics	Prenatal Screening Test		
	1st Trimester Combined Test	2nd Trimester Triple Screen	Non-Invasive Prenatal Testing
Number of Studies	6	9	4
Number of Patients	95,159	1,385,296	1491
Number of Cases	37	290	30
Median prevalence per 10,000 pregnancies (25th–75th percentile range)	4.2 (2.5–9.3)	3.2 (2.5–6.1)	397.5 (206.9–583.1)
Observed Detection Rates from Included Studies (Minimum-Maximum Range)	0%–100%	0%–100%	75%–100%
Observed False Positive Rates from Included Studies (Minimum-Maximum Range)	4.3%–7.2%	5.1%–26.1%	0%–0.2%
Pooled Detection Rate	70.1% (51.8–83.7)	77.2% (59.9–88.5)	92.2% (91.6–92.8)
Pooled False Positive Rate	5.4% (4.7–6.3)	9.3% (6.7–12.8)	0.1% (0.11–0.12)

With the exception of NIPT, none of the other screening tests involved a specific risk cut-off to identify patients at increased risk of carrying a fetus with Turner syndrome. Turner syndrome is considered an incidental finding amongst patients who screen positive for trisomy 13, 18 or 21 with the first trimester combined test and the second trimester triple and quadruple screens. This has implications for the interpretation of test results in a clinical setting.

NIPT had the best overall test performance with a detection rate of 92.2% and a false positive rate of <0.1% (Table 4). A significant difference was not observed between the first trimester combined test and the second trimester triple screen for detection rate, although the first trimester combined test had a significantly lower false positive rate (*p* < 0.05) (Table 4). The single study that used the second trimester quadruple screen had an observed detection rate of 66.7% and a false positive rate of 9.6% [76].

### 3.4. Triploidy

Finally, 11 studies provided data on triploidy: 7 of which used the first trimester combined test [19,20,23,24,27,30,51], 3 utilized the second trimester triple screen [32,33,35] and 1 used the second trimester quadruple screen [76]. Triploidy was an incidental finding amongst women who were screen positive for trisomy 13, 18 or 21. This has implications for the interpretation of test results in a clinical setting and how patients should be counseled following a positive screening test.

Seven studies, representing 93,796 women and 15 affected fetuses, contained data on the first trimester combined screen and could be pooled using diagnostic meta-analysis [19,20,23,24,27,30,51]. These studies had a pooled detection rate of 100.0% (99.9–100.0) and a pooled false positive rate of 6.3% (4.9–8.0) (Appendix B). Comparable results were also observed for the other screening tests, even though sufficient data were not available to derive pooled estimates. Observed detection rates ranged from 98.1% to 100% with observed false positive rates of 2.6%–10.6% for the second trimester triple screen [32,33,35]; while the single study that used the quadruple screen reported a detection rate of 100% and a false positive rate of 9.6%.

## 4. Discussion

The results of this systematic review and diagnostic meta-analysis confirm the general consensus that for all conditions, NIPT is a superior test in terms of detection rate and false positive rate than other screening tests for aneuploidies, with the caveat that most studies have been performed in high risk populations. However, NIPT is subject to a higher rate of test failures than other prenatal screening tests and currently only provides results for a limited number of aneuploidies. The dramatically higher median prevalence of aneuploidies used in studies of NIPT to date has important implications on test performance in a low-risk setting. While this is unlikely to impact the overall detection rate and false positive rate, positive predictive values (odds of being affected given a positive result) are particularly sensitive to the prevalence of the condition being studied [80,81]. Additionally, pooled results for NIPT did not display 100% detection rates for any condition, indicating the importance of confirmatory invasive testing. Furthermore, due to the high cost of NIPT, many centers continue to utilize other forms of prenatal screening. Nonetheless, having data on pooled detection rates and false positives rates may be helpful when counseling patients.

Multiple studies have indicated that up to half of the chromosomal anomalies identified through invasive testing for abnormal prenatal screening results or increased maternal age are not autosomal aneuploidies [15,82,83]. Several authors have expressed concern that moving away from serum and ultrasound based screening to prenatal screening based exclusively on NIPT might miss the detection of rare chromosomal anomalies [5,73,84]. Furthermore, extreme levels of serum analytes are associated with adverse obstetrical outcomes and may be useful to help triage patients into higher levels of prenatal care [85,86,87,88]. Contingent screening with NIPT might provide a compromise in terms of maintaining the benefits of existing prenatal screening programs, while reducing the number of women who proceed onto invasive testing, but the current costs of NIPT make this prohibitive to implement in many population-based screening programs. While the costs of NIPT will likely decrease over time, the current cost of NIPT is $795 (Canadian dollars) in contrast to $303 for the first trimester combined test and $15 for the second trimester triple screen [89,90].

Current clinical practice guidelines recommend that prenatal screening should be offered through an informed consent process; in particular, the Society of Obstetricians and Gynecologists of Canada has issued counseling recommendations specifically detailing that all women who are offered prenatal screening should be told that all women have some risk of having a fetus affected by trisomy 21, 18, or 13 [91]. We suggest additional pre-screening counseling recommendations that women should be informed that, while prenatal screening tests have been developed to specifically target the detection of trisomy 21, 18, and 13, other chromosomal anomalies and obstetrical risks may be detected by the screen as well. In particular, it would be worth discussing sex chromosome aneuploidy and triploidy in light of available data, but some mention of the breadth of unexpected results would enhance the informed consent process.

This study has limitations. The search specifically excluded terms related to trisomy 21—this was done on purpose to restrict the number of abstracts identified and because the goal of this review was to look at chromosomal anomalies other than trisomy 21. This may have resulted in some relevant articles not being identified in the initial search; however, the reference lists of accepted articles were hand searched to identify other relevant articles. The quality of the pooled results is a direct reflection of the data included in the original articles; the authors of many studies made the assumption that false negative cases would be brought to their attention through cytogenetic databases or birth certificates and did not actively follow-up all women screened. Additionally, the raw data on true positives, false positives, false negatives and true negatives sometimes had to be back-calculated based on reported detection and false positive rates; this may have resulted in some minor inaccuracies. The model used to generate pooled detection and false positive rates does not specifically account for differences in cut-off values or the specific screening algorithm used to define a positive or negative screening test [14]. However, this is reflected in the different estimates of sensitivity and specificity obtained from individual studies and the heterogeneity of these estimates is directly modeled and used to derive the pooled estimates [14]. Multiple between-study differences were observed in terms of the maternal age distribution, inclusion of pregnancies that ultimately resulted in spontaneous abortion and of multiple gestation pregnancies, test uniformity (specifically related to the use of free β hCG *vs.* total hCG), and test quality standards (specifically related to the use of nuchal translucency). While a random effects model was used to derive pooled estimates, this model addresses statistical heterogeneity, not clinical heterogeneity in the underlying populations. Finally, the search was limited to English language articles which may limit the generalizability of the results. However, the included studies came from North America, Europe, Australia and Asia, indicating wide geographic coverage.

## 5. Conclusions

In conclusion, while prenatal screening tests are often described to patients in terms of trisomy 21, they do (to varying degrees) identify other chromosomal anomalies. Providing this information to patients prior to screening can help them make an informed choice about accessing prenatal screening and, in some contexts, which screening test is preferable to them.

## Figures and Tables

**Appendix Figure B1:**
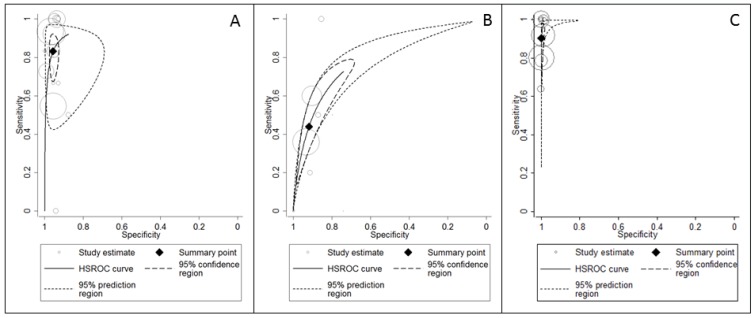
Summary Receiver Operator Characteristic (SROC) Curves for the detection of trisomy 13 using (**A**) first trimester combined test; (**B**) second trimester triple screen; and (**C**) non-invasive prenatal testing.

**Appendix Figure B2:**
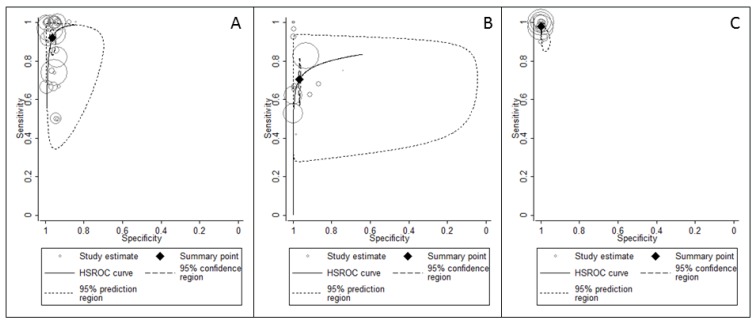
Summary Receiver Operator Characteristic (SROC) Curves for the detection of trisomy 18 using (**A**) first trimester combined test; (**B**) second trimester triple screen; and (**C**) non-invasive prenatal testing.

**Appendix Figure B3:**
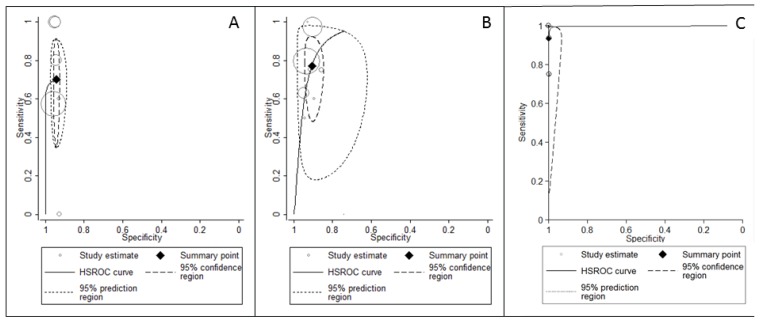
Summary Receiver Operator Characteristic (SROC) Curves for the detection of 45,X using (**A**) first trimester combined test; (**B**) second trimester triple screen; and (**C**) non-invasive prenatal testing.

**Appendix Figure B4:**
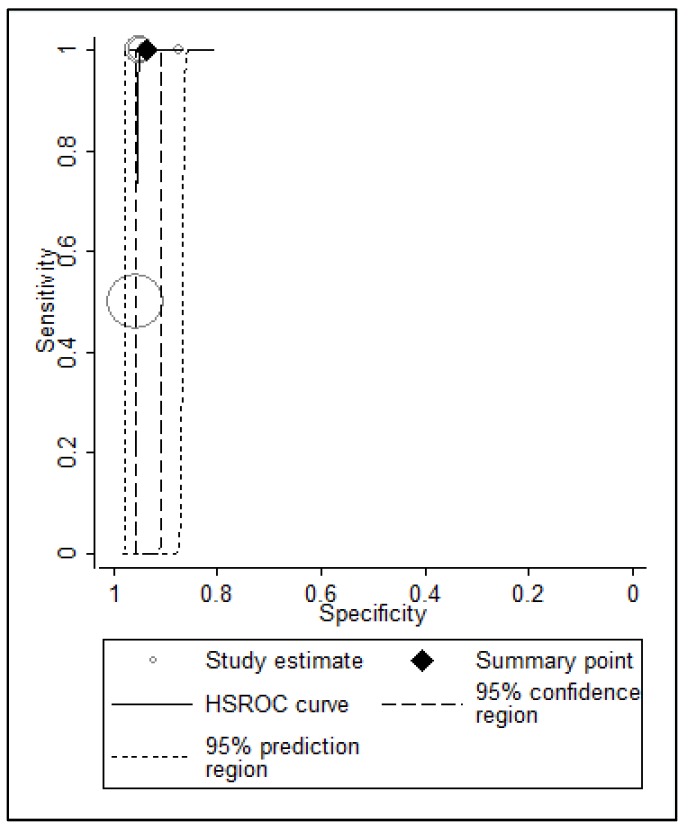
Summary Receiver Operator Characteristic (SROC) Curves for the detection of triploidy using the first trimester combined test.

## Appendix

**Appendix A:** Included studies.

Study	Screening Test	*N*	Detection Rate	False Positive Rate	Cut-Off to Define a Positive Screening Test	Location
*Trisomy 13*						
Alamillo 2013 [15]	First Trimester Combined Test	23,329	100.0	6.3	T21: 1/300 T13/T18: 1/150	USA
Berktold 2013 [29]	First Trimester Combined Test	14,862	83.3	4.7		Germany
Hormansdorfer 2009 [16]	First Trimester Combined Test	2202	0	5.8	1/230	Germany
Kagan 2008 [17]	First Trimester Combined Test	56,954	93.4	3.1		UK
Karadozov-Orlic 2012 [18]	First Trimester Combined Test	4172	81.8	5.3		Serbia
Marttala 2011 [19]	First Trimester Combined Test	56,076	54.5	4.5	T21: 1/250 T13/T18: 1/200	Finland
Merz 2008 [28]	First Trimester Combined Test	40,802	92.3	5.0		Germany
Ochshorn 2001 [26]	First Trimester Combined Test	1408	66.7	7.1		Israel
Orlandi 1997 [20]	First Trimester Combined Test	2010	50.0	12.5		Italy
Scott 2004 [30]	First Trimester Combined Test	2053	100.0	7.2		Australia
Sorensen 2011 [21]	First Trimester Combined Test	19,694	72.7	1.0	T21: 1/300 T13/T18: 1/150	Denmark
Spencer 2000 [22]	First Trimester Combined Test	989	83.3	0.1		England
Spencer 2000 [27]	First Trimester Combined Test	3762	100.0	6.7	T13/T18/T21: 1/300	England
Spencer 2003 [23]	First Trimester Combined Test	11,105	100.0	5.2	T13/T18/T21: 1/300	England
Stenhouse 2004 [24]	First Trimester Combined Test	5084	100.0	6.2	T13/T18/T21: 1/250	Scotland
Valinen 2012 [25]	First Trimester Combined Test	1000	66.7	4.6	T13/T18: 1/200	Finland
Benn 1996 [31]	Second Trimester Triple Screen	26,364	20.0	8.7	T21: 1/270 T18: AFP ≤ 0.75 MoM, Hcg ≤ 0.55 MoM, uE3 ≤ 0.6 MoM	USA
Burton 1993 [32]	Second Trimester Triple Screen	8233	0	0.2	T21: 1/270 T18: AFP ≤ 0.7 MoM, hCG ≤ 0.5 MoM, uE3 ≤ 0.55 MoM	USA
Kazerouni 2009 [33]	Second Trimester Triple Screen	752,686	36.0	6.6	T21: 1/190 T18: 1/100	USA
Onda 2000 [34]	Second Trimester Triple Screen	32,925	100.0	14.5	T21: 1/295 T18: 1/100	Japan
Summers 2003 [35]	Second Trimester Triple Screen	423,895	60.0	9.7	T21: 1/385 at term T18: 1/100	Canada
Suzumori 1997 [37]	Second Trimester Triple Screen	1078	50.0	20.3	T21: 1/299	Japan
Wenstrom 1995 [36]	Second Trimester Triple Screen	1423	0	26.1	T21: 1/190 T18: AFP ≤ 0.75 MoM, hCG ≤ 0.55 MoM, uE3 ≤ 0.6 MoM	USA
Wortelboer 2008 [38]	Second Trimester Triple Screen	30,290	50.0	13.1	T21: 1/200 T18: 1/200	Netherlands
Ashoor 2013 [39]	Non-invasive prenatal testing	156	63.6	0		England
Ashoor 2013 [39]	Non-invasive prenatal testing	1949	80.0	0.1		England and USA
Bianchi 2012 [40]	Non-invasive prenatal testing	532	78.6	0		USA
Chen 2011 [41]	Non-invasive prenatal testing	392	100.0	1.1		Hong Kong, Netherlands, UK
Jiang 2012 [42]	Non-invasive prenatal testing	903	100.0	0		China
Lau 2012 [43]	Non-invasive prenatal testing	108	100.0	0		Japan
Palomaki 2012 [44]	Non-invasive prenatal testing	1688	91.7	1.0	z score ≥ 3	Argentina, Australia, Canada, Czech Republic, Hungary, Ireland, Italy, Spain, USA
Sehnert 2011 [45]	Non-invasive prenatal testing	47	100.0	0	>2.5 standard deviations of the mean	USA
Sehnert 2011 [45]	Non-invasive prenatal testing	65	100.0	1.6	>2.5 standard deviations of the mean	USA
*Trisomy 18*															
Alamillo 2013 [15]	First trimester combined test	23,329	100.0	6.3	T21: 1/300 T13/18: 1/150	USA
Berktold 2013 [29]	First trimester combined test	14,862	100.0	4.7		Germany
Borrell 2004 [46]	First trimester combined test	2765	75.0	3.3	T21: 1/250	Spain
Breathnach 2007 [47]	First trimester combined test	35,974	82.1	6.0	T21: 1/150 T18: 1/100	USA
Centini 2005 [48]	First trimester combined test	408	100.0	15.8		Italy
Chou 2009 [49]	First trimester combined test	10,811	50.0	5.4	T21: 1/270	Taiwan
Dhaifalah 2006 [50]	First trimester combined test	686	100.0	5.1	1/250	Czech Republic
Gaffari 2012 [51]	First trimester combined test	13,706	100.0	4.7	1/300	Iran
Guanciali-Franchi 2011 [52]	First trimester combined test	7292	66.7	4.2		Italy
Hormansdorder 2009 [16]	First trimester combined test	2202	50.0	5.7	1/230	Germany
Jacques 2007 [53]	First trimester combined test	15,243	66.7	0.4	T21: 1/300 T18: 1/250	Australia
Kagan 2008 [17]	First trimester combined test	56,954	96.7	2.4		UK
Karadzov-Orlic 2012 [18]	First trimester combined test	4172	85.7	5.3		Serbia
Krantz 2000 [54]	First trimester combined test	5718	100.0	0.8	T18: 1/150	USA
Martinez-Morillo 2012 [55]	First trimester combined test	18,801	100.0	0.4	1/250	Spain
Marttala 2011 [19]	First trimester combined test	56,076	74.1	4.5	T21: 1/250 T13/18: 1/200	Finland
Merz 2008 [28]	First trimester combined test	40,802	94.1	5.0		Germany
Ochshorn 2001 [26]	First trimester combined test	1408	66.7	7.1		Israel
Orlandi 1997 [20]	First trimester combined test	2010	100.0	12.3		Italy
Perni 2006 [56]	First trimester combined test	4615	100.0	1.0		USA
Scott 2004 [30]	First trimester combined test	2053	100.0	7.2		Australia
Sorensen 2011 [21]	First trimester combined test	19,694	91.3	1.5	T21: 1/300 T13/18: 1/150	Denmark
Spencer 2000 [27]	First trimester combined test	3762	100.0	6.6	T13/18/21: 1/300	England
Spencer 2003 [23]	First trimester combined test	11,105	100.0	5.1	T13/18/21: 1/300	England
Spencer 2007 [57]	First trimester combined test	521	96.2	1.3	T21: 1/300 T13/18: 1/100	UK
Stenhouse 2004 [24]	First trimester combined test	5084	100.0	6.1	T13/18/21: 1/250	Scotland
Tsai 2001 [58]	First trimester combined test	1514	50.0	6.3	T21: 1/400	Taiwan
Tul 1999 [59]	First trimester combined test	997	90.0	1.1		England
Valinen 2012 [25]	First trimester combined test	1000	73.7	4.6	T13/18: 1/200	Finland
Wapner 2003 [60]	First trimester combined test	8216	100.0	2.0	T21: 1/270 T18:1/150	USA
Barkai 1993 [61]	Second trimester triple screen	5502	66.7	0.3	T21: 1/300 T18: 1/100	Israel
Benn 1996 [31]	Second trimester triple screen	26,364	62.5	8.6	T21: 1/270 T18: AFP ≥ 0.75 MoM, hCG ≤ 0.55 MoM, uE3 ≤ 0.6 MoM	USA
Benn 1999 [62]	Second trimester triple screen	41,565	92.3	0.4	T21: 1/270 T18: 1/100	USA
Burton 1993 [32]	Second trimester triple screen	8233	100.0	0.2	T21: 1/270 T18: AFP ≥ 0.7 MoM, hCG ≤ 0.5 MoM, uE3 ≤ 0.55 MoM	USA
Hogge 2001 [63]	Second trimester triple screen	45,145	66.7	0.5	1/100	USA
Kazerouni 2009 [33]	Second trimester triple screen	752,686	82.5	6.6	T21: 1/190 T18: 1/100	USA
Kellner 1995 [64]	Second trimester triple screen	8649	66.7	0.2	T18: AFP ≥ 0.75 MoM, hCG ≤ 0.55 MoM, uE3 ≤ 0.6 MoM	USA
Kishida 2000 [65]	Second trimester triple screen	1055	60.0	35.7	T21: 1/299 or AFP ≥ 2.5 MoM	Japan
McDuffie 1996 [66]	Second trimester triple screen	6197	100.0	0.2	T21: 1/295 T18: AFP ≥ 0.75 MoM, hCG ≤ 0.55 MoM, uE3 ≤ 0.6 MoM	USA
Meier 2003 [67]	Second trimester triple screen	382,598	62.2	0.2	1/30 (2nd Trimester) or 1/100 at term	Canada
Onda 2000 [34]	Second trimester triple screen	32,925	96.4	0.5	T21: 1/295 T18: 1/100	Japan
Palomaki 1995 [68]	Second trimester triple screen	10,620	65.2	0.2		USA
Summers 2003 [35]	Second trimester triple screen	423,895	52.8	0.2	T21: 1/385 (at term) T18: 1/100	Canada
Suzumori 1997 [37]	Second trimester triple screen	1078	0	20.4	T21: 1/299	Japan
Wenstrom 1995 [36]	Second trimester triple screen	1423	75.0	25.9	T21: 1/190 T18: AFP ≥ 0.75 MoM, hCG ≤ 0.55 MoM, uE3 ≤ 0.6 MoM	USA
Wenstrom 1997 [69]	Second trimester triple screen	5327	41.7	1.8	T21: 1/190 T18: AFP ≥ 0.75 MoM, hCG ≤ 0.55 MoM, uE3 ≤ 0.6 MoM	USA
Wortelboer 2008 [38]	Second trimester triple screen	30,290	67.9	13.1	T21: 1/250 T18: 1/200	Netherlands
Breathnach 2007 [47]	Second trimester quadruple screen	35,120	100.0	8.9	T21: 1/300 T18: 1/100	USA
Jacques 2006 [75]	Second trimester quadruple screen	16,607	44.4	0.5	T21: 1/250 T18: 1/200	Australia
Kwon 2012 [76]	Second trimester quadruple screen	9435	100.0	9.6	T21: 1/270 T18: 1/100	Korea
Ashoor 2012 [74]	Non-invasive prenatal testing	397	980	0		UK
Bianchi 2012 [40]	Non-invasive prenatal testing	532	97.2	0		USA
Chen 2011 [41]	Non-invasive prenatal testing	392	91.9	2.0		Hong Kong, Netherlands, UK
Dan 2012 [70]	Non-invasive prenatal testing	3000	100.0	0.03		China
Jiang 2012 [42]	Non-invasive prenatal testing	903	100.0	0.1		China
Lau 2012 [43]	Non-invasive prenatal testing	108	90.0	0		Japan
Nicolaides 2012 [73]	Non-invasive prenatal testing	1949	100.0	0.	Risk ≥1%	UK
Norton 2012 [71]	Non-invasive prenatal testing	3080	97.4	0.1	1/100	USA, Netherlands, Sweden
Palomaki 2012 [44]	Non-invasive prenatal testing	1971	100.0	0.3	z score ≥ 3	Argentina, Australia, Canada, Czech Republic, Hungary, Ireland, Italy, Spain, USA
Sehnert 2011 [45]	Non-invasive prenatal testing	65	100.0	1.6	>2.5 standard deviations of the mean	USA
Sparks 2012 [72]	Non-invasive prenatal testing	167	100.0	0.6		USA
Sparks 2012 [72]	Non-invasive prenatal testing	163	100.0	0		USA
Benn 2007 [77]	Integrated screening	1203	100.0	7.3		USA
Guanciali-Franchi 2011 [52]	Integrated screening	7292	100.0	3.7	1/250	Italy
*45,X*															
Chou 2009 [49]	First trimester combined test	10,811	80.0	5.4	T21: 1/270	Taiwan
Ghaffari 2012 [51]	First trimester combined test	13,706	100.0	4.7	1/300	Iran
Marttala 2011 [19]	First trimester combined test	56,076	57.1	4.3	T21: 1/250 T13/T18: 1/200	Finland
Ochshorn 2001 [26]	First trimester combined test	1408	60.0	7.1		Israel
Scott 2004 [30]	First trimester combined test	2053	0	7.2		Australia
Spencer 2003 [23]	First trimester combined test	11,105	100.0	5.2	T13/T18/T21: 1/300	England
Benn 1996 [31]	Second trimester triple screen	26,364	75.0	8.7	T21: 1/270 T18: AFP ≤ 0.75 MoM, hCG ≤ 0.55 MoM, uE3 ≤ 0.6 MoM	USA
Burton 1993 [32]	Second trimester triple screen	8233	60.0	10.6	T21: 1/270 T18: AFP ≤ 0.7 MoM, hCG ≤ 0.5 MoM, uE3 ≤ 0.55 MoM	USA
Kazerouni 2009 [33]	Second trimester triple screen	752,686	79.4	6.6	T21: 1/190 T18: 1/100	USA
McDuffie 1996 [66]	Second trimester triple screen	6197	50.0	5.6	T21: 1/295 T18: AFP ≤ 0.75 MoM, hCG ≤ 0.55 MoM, uE3 ≤ 0.6 MoM	USA
Onda 2000 [34]	Second trimester triple screen	32,925	75.0	14.5	T21: 1/295 T18: 1/100	Japan
Ruiz 1999 [78]	Second trimester triple screen	130,595	63.2	5.1	T21: 1/225	USA
Summers 2003 [35]	Second trimester triple screen	423,895	97.3	9.7	T21: 1/385 (at term) T18: 1/100	Canada
Valerio 1996 [79]	Second trimester triple screen	2978	100.0	7.1	T21: 1/270	Italy
Wenstrom 1995 [36]	Second trimester triple screen	1423	0	26.1	T21: 1/190 T18: AFP ≤ 0.75 MoM, hCG ≤ 0.55 MoM, uE3 ≤ 0.6 MoM	USA
Kwon 2012 [76]	Second trimester quadruple screen	9435	66.7	9.6	T21: 1/270 T18: 1/100	Korea
Bianchi 2012 [40]	Non-invasive prenatal testing	433	93.8	0.2		USA
Jiang 2012 [42]	Non-invasive prenatal testing	903	75.0	0.2		China
Lau 2012 [43]	Non-invasive prenatal testing	108	100.0	0		Japan
Sehnert 2011 [45]	Non-invasive prenatal testing	47	100.0	0	>2.5 standard deviations of the mean	USA
*Triploidy*						
Ghaffari 2012 [51]	First trimester combined test	13,706	100.0	4.8	1/300	Iran
Marttala 2011 [19]	First trimester combined test	56,076	50.0	4.3	T21: 1/250 T13/T18: 1/200	Finland
Orlandi 1997 [20]	First trimester combined test	2010	100.0	12.4		Italy
Scott 2004 [30]	First trimester combined test	2053	100.0	7.2		Australia
Spencer 2000 [27]	First trimester combined test	3762	100.0	6.7	T13/T18/T21: 1/300	England
Spencer 2003 [23]	First trimester combined test	11,105	100.0	5.2	T13/T18/T21: 1/300	England
Stenhouse 2004 [24]	First trimester combined test	5084	100.0	6.2	T13/T18/T21: 1/250	Scotland
Burton 1993 [32]	Second trimester triple screen	8233	100.0	10.6	T21: 1/270 T18: AFP ≤ 0.7 MoM, hCG ≤ 0.5 MoM, uE3 ≤ 0.55 MoM	USA
Kazerouni 2009 [33]	Second trimester triple screen	752,686	98.1	6.6	T21: 1/190 T18: 1/100	USA
Summers 2003 [35]	Second trimester triple screen	423,895	100.0	9.7	T21: 1/385 at term T18: 1/100	Canada
Kwon 2012 [76]	Second trimester quadruple screen	9435	100.0	9.6	T21: 1/270 T18: 1/100	Korea

## Appendix B—Summary Receiver Operator Characteristic (SROC) Curves

The Summary Receiver Operator Characteristic (SROC) curves below plot the sensitivity (detection rate) of a given test against 1-specificity (false positive rate) of a given test for detecting the condition of interest [13]. Circles represent the point estimate from included studies; the size of the circle is related to the sample size of the study [13]. Diamonds represent the pooled detection rate and false positive rate [13]. The dashed lines represent the 95% confidence region surrounding the pooled effect estimate, while the dotted lines represent the 95% prediction region that would encompass the true detection rate and false positive rates in future studies [13]. The HSROC curve represents the summary receiver operator characteristic curve obtained from the model [13].
